# Differential gene expression analysis of palbociclib-resistant TNBC via RNA-seq

**DOI:** 10.1007/s10549-021-06127-5

**Published:** 2021-02-18

**Authors:** Lilibeth Lanceta, Nadiia Lypova, Conor O’Neill, Xiaohong Li, Eric Rouchka, Jason Chesney, Yoannis Imbert-Fernandez

**Affiliations:** 1 Department of Medicine, School of Medicine, University of Louisville, Louisville, KY 40202 USA; 2 College of Medicine, University of Kentucky, Lexington, KY 40506 USA; 3 Department of Anatomical Sciences and Neurobiology, Bioinformatics Core, University of Louisville, Louisville, KY 40202 USA; 4 Department of Computer Engineering and Computer Science, University of Louisville, Louisville, KY 40292 USA; 5 James Graham Brown Cancer Center School of Medicine, University of Louisville, Louisville, KY 40202 USA

**Keywords:** Palbociclib, TNBC, CDK4/6 inhibitors, Therapy resistance, Metabolism reprogramming

## Abstract

**Purpose:**

The management of triple-negative breast cancer (TNBC) remains a significant clinical challenge due to the lack of effective targeted therapies. Inhibitors of the cyclin-dependent kinases 4 and 6 (CDK4/6) are emerging as promising therapeutic agents against TNBC; however, cells can rapidly acquire resistance through multiple mechanisms that are yet to be identified. Therefore, determining the mechanisms underlying resistance to CDK4/6 inhibition is crucial to develop combination therapies that can extend the efficacy of the CDK4/6 inhibitors or delay resistance. This study aims to identify differentially expressed genes (DEG) associated with acquired resistance to palbociclib in ER− breast cancer cells.

**Methods:**

We performed next-generation transcriptomic sequencing (RNA-seq) and pathway analysis in ER− MDA-MB-231 palbociclib-sensitive (231/pS) and palbociclib-resistant (231/pR) cells.

**Results:**

We identified 2247 up-regulated and 1427 down-regulated transcripts in 231/pR compared to 231/pS cells. DEGs were subjected to functional analysis using Gene Ontology (GO) and the KEGG database which identified many transduction pathways associated with breast cancer, including the PI3K/AKT, PTEN and mTOR pathways. Additionally, Ingenuity Pathway Analysis (IPA) revealed that resistance to palbociclib is closely associated with altered cholesterol and fatty acid biosynthesis suggesting that resistance to palbociclib may be dependent on lipid metabolic reprograming.

**Conclusion:**

This study provides evidence that lipid metabolism is altered in TNBC with acquired resistance to palbociclib. Further studies are needed to determine if the observed lipid metabolic rewiring can be exploited to overcome therapy resistance in TNBC.

**Supplementary Information:**

The online version contains supplementary material available at 10.1007/s10549-021-06127-5.

## Introduction

Triple-negative breast cancer (TNBC) encompasses an heterogenous subtype of breast cancer that is histologically defined by the lack of estrogen receptor (ER), progesterone receptor (PR) and HER-2/neu overexpression [1, 2]. Although this breast cancer subtype only accounts for only 10–20% of all breast cancers, it is associated with poor prognosis due to the high risk of distant recurrence [3]. Because of the lack of hormone receptors and HER-2/neu amplification, management of TNBC has been mainly limited to surgery and chemotherapy. More recently, a subset of TNBC patients are now treated with biomarker-driven therapies such as PARP inhibitors or platinum agents and immune checkpoint inhibitors (Keytruda, Tecentriq) in PD-L1-positive TNBC. Additionally, a new class of potent anticancer drugs such as antibody–drug conjugates (Trodelvy) are now FDA approved for TNBC [4]. Despite these promising novel therapies, their ability to improve 5-year relative survival is still limited. Compared with ER-positive (ER+) breast cancer patients, the 5-year relative survival rates for localized, regional and distant TNBC patients are 91%, 65% and 11.5%, respectively, vs 100%, 89.9% and 30.4% [5]. This disparity in survival compared to ER+ breast cancer is largely due to the lack of targeted therapies against TNBC [6]. Thus, effective treatments against TNBC are urgently needed to improve the overall survival of these patients.

Aberrant activation of the cyclin D1-CDK4/6-retinoblastoma (Rb) pathway is hallmark of breast cancer that led to the development of CDK4/6 inhibitors [7–11]. Three selective CDK4/6 inhibitors (palbociclib, abemaciclib, and ribociclib) have been FDA approved for the treatment of metastatic ER-positive (ER+) breast cancer patients in combination with endocrine therapy given their proved ability to increase progression-free survival [12]. TNBC was initially considered a poor candidate for CDK4/6 inhibition given that Rb loss and high cyclin E expression are often observed in these tumors [13, 14]. However, recent studies have demonstrated that TNBC expressing the Rb protein are sensitive to CDK4/6 inhibition providing a strong rationale to extend the use of CDK4/6 inhibitors to TNBC [15–20]. As an example, simultaneous inhibition of CDK4/6 and PI3K was shown to be highly effective both in vivo and in vitro against various Rb-positive preclinical TNBC models, including patient derived xenografts (PDX) [18]. Additionally, the triple combination of CDK4/6, PI3K and immune-checkpoint blockage demonstrated long-lasting anti-tumor activity against TNBC in vivo [18]. Another important study demonstrated that CDK4/6 inhibition blocks the tumor metastasis potential of multiple preclinical models of TNBC without affecting primary tumor growth [19]. Taken together, these observations provide strong rationale for the potential use of CDK4/6 inhibitor for the treatment of TNBC. Consequently, multiple trials aiming to evaluate the utility of CDK4/6 as single agents or in combination with other targeted therapies against TNBC are currently ongoing (ClinicalTrials.gov; NCT03090165, NCT02978716, NCT03979508, NCT03519178).

CDK4/6 inhibition is emerging as a promising therapeutic approach against TNBC; however, we anticipate that resistance will arise and become a significant clinical challenge for TNBC patients given the known development of resistance observed in nearly all ER+ patients receiving this therapy [21]. Thus, the identification of mechanisms of resistance to CDK4/6 blockage will be crucial for the further development of this treatment modality in TNBC patients. The major goal of this study was to identify actionable targets and pathways driving resistance to CDK4/6 inhibition in TNBC through transcriptomic analysis. Using MDA-MB-231 cells as a model of TNBC, we performed next-generation transcriptomic RNA sequencing (RNA-seq) on palbociclib-resistant MDA-MB-231 (231/pR) and their isogenic palbociclib-sensitive counterpart (231/pS). Differential gene expression and pathway analysis indicated that resistance to palbociclib involves many canonical pathways including aryl hydrocarbon receptor, immune responses and signal transduction pathways such as PI3K, PTEN and mTOR signaling. Notably, our analysis showed that cholesterol and lipid biosynthesis were uniquely enriched in palbociclib-resistant cells compared to palbociclib-sensitive. These studies identified novel druggable candidates and pathways that may be able to prevent or alleviate resistance to palbociclib in TNBC patients.

## Materials and methods

### Cell culture, generation of palbociclib-resistant cells

MDA-MB-231 (HTB-26) cells were purchased from the American Type Culture Collection (ATCC) and maintained at 37 °C with 5% CO_2_. MDA-MB-231 cells were cultured in IMEM (Corning) supplemented with 10% fetal bovine serum (FBS, Invitrogen). MFM-223 cells were purchased from SIGMA and maintained at 37 °C with 5% CO_*2*_. MFM-223 cells were cultured in MEM (Corning) supplemented with 10% fetal bovine serum (FBS, Invitrogen). Palbociclib-resistant MDA-MB-231 and MFM-223 cells were established by culturing in media containing palbociclib (0.1–4 μM). Drug was replenished every 3 days. Cells were subcultured every 1–2 weeks with 25% increments in drug concentration. The resistant cells were established after 4–6 months and maintained in the presence of 1 μM palbociclib. Cells were authenticated by the short tandem repeat (STR) assay (Genetica).

### RNA extraction and next-generation sequencing

Total RNA was extracted from 231/pS and 231/pR cells using the RNeasy kit (Qiagen) following the manufacturer’s instructions (three independent replicates per cell line). Libraries were prepared simultaneously using the TruSeq Stranded mRNA LT Sample Prep Kit- Set A (Cat# RS-122-2101) with poly-A enrichment. Sequencing was performed on the University of Louisville Center for Genetics and Molecular Medicine’s (CGeMM) Illumina NextSeq 500 using the NextSeq 500/550 1 × 75 cycle High Output Kit v2 (Cat# FC-404-2005). A second run was performed on all samples to achieve an average of 45 million reads per sample.

### DEG analysis

The resulting samples were downloaded from Illumina’s BaseSpace [22] (https://basespace.illumina.com/). Sequences were directly aligned to the Homo sapiens hg38 reference genome assembly (hg38.fa) using tophat2 (version 2.0.13), generating alignment files in bam format. DEG were identified for the pairwise comparison 231/pS vs 231/pR using the tuxedo suite programs including cufflinks-cuffdiff2 (VERSION2.2.1). A *q*-value cutoff ≤ 0.05 with |log_2_FC| ≥ 1 and gene expression > 1 in at least one replicate was used to determine differential expression. RNAseq data available (GEO accession number GSE130437). Gene Ontology Biological Processes (GO:BP) and KEGG Pathways analysis was performed using CategoryCompare [23].

### In silico ingenuity network analysis

Pathway and biological processes analysis of all differentially expressed genes was performed using Ingenuity Pathway Analysis (Qiagen).

### Western blot analysis

Cells were treated with either vehicle control (H_2_O) or 500 nM palbociclib (PD-0332991, Selleckchem) for 24 h prior to protein isolation. Whole cell lysates were harvested using RIPA buffer (ThermoFisher) supplemented with protease and phosphatase inhibitors. Protein concentration was determined using the BCA protein assay kit (ThermoFisher) following the manufacturer’s instructions. Proteins were separated on 4–20% Mini PROTEAN TGX gels under reducing conditions and transferred to Immun-Blot PVDF membranes (Bio-Rad). The membranes were blocked with 5% BSA or 5% nonfat milk in TBS-T (0.1% Tween20) and immunoblotted with the indicated antibodies. Antibody against DGKG gamma (PA5-97658) was purchased from Invitrogen. SREBP1 (sc-365513) and MVK (sc-390669) antibodies were purchased from Santa Cruz Biotechnology. Antibody against FASN (no. 3180) was purchased from Cell Signaling. Beta-actin (A2228) antibody was purchased from Sigma. Antibodies against HRP-conjugated goat anti-rabbit (Invitrogen) and anti-mouse IgG (Sigma) were used as secondary antibodies. Amersham ECL Prime Western blotting detection reagent (GE Healthcare) was used to detect immunoreactive bands. The bands were visualized on autoradiography film BX (MidSci). Quantitative densitometry was performed with UN-SCAN-IT (Silk scientific, version 6.3), signal density was normalized to the corresponding beta-actin loading control.

## Results

### Transcriptomic profiling of ER− palbociclib-resistant cells

**Fig. 1:**
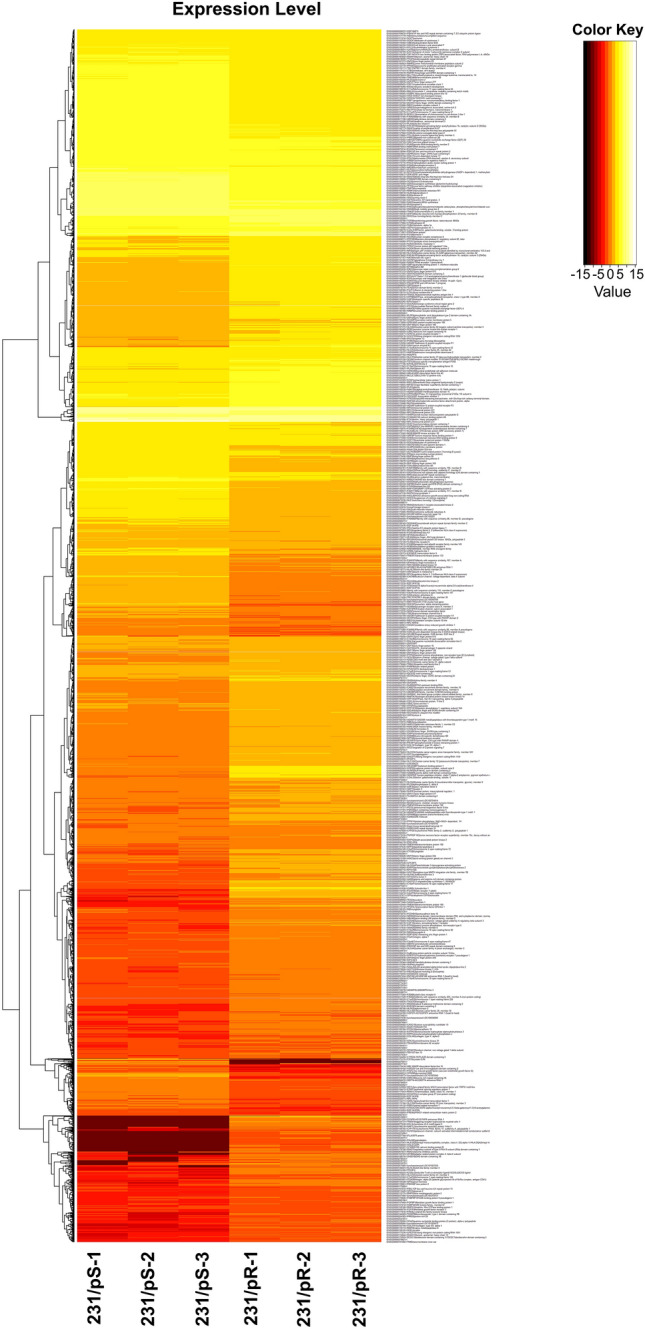
Differential expression heatmap visualization of ER− 231/pS compared to 231/pR cells. Next-generation transcriptomic RNA sequencing (RNA-seq) was performed and the raw expression of genes is shown as a heatmap. Replicate samples are clustered. Red and yellow indicate lower and higher gene expression, respectively

The development and characterization of palbociclib-resistant, 231/pR cells, and its isogenic parental cell line (231/pS) was previously described by our group [24]. We performed gene expression profiling in 231/pS and 231/pR cells with the goal of characterizing transcriptional alterations upon the development of resistance to palbociclib. Differential gene expression was defined as |log_2_FC| ≥ 1 with a *q*-value cutoff ≤ 0.05 between the resistant and its isogenic sensitive cell line. Hierarchical clustering based on differentially expressed RNA transcripts revealed a distinct transcriptomic profile in 231/pR cells compared to 231/pS (Fig. 1), with 2247 upregulated genes and 1427 downregulated transcripts identified in 231/pR cells. The top 20 upregulated and downregulated genes in 231/pR cells compared to 231/pS cells are shown in Table 1.

**Table 1:** Top 20 up-regulated and down-regulated genes between 231/pS and 231/pR ranked by *p*-value (*p*_val_ ≤ 0.05; *q*_val_ ≤ 0.05; |log_2_FC|≥ 1)

Ensembl ID	Gene symbol|Description	Log2FC	*p*-value	*Q*-value
Up-regulated				
ENSG00000058866	DGKG|diacylglycerol kinase, gamma 90 kDa	19.8941	5.00E−05	0.000208859
ENSG00000079393	DUSP13|dual specificity phosphatase 13	19.8941	5.00E−05	0.000208859
ENSG00000099260	PALMD|palmdelphin	19.8941	5.00E−05	0.000208859
ENSG00000105472	CLEC11A|C-type lectin domain family 11, member A	19.8941	5.00E−05	0.000208859
ENSG00000108691	CCL2|chemokine (C–C motif) ligand 2	19.8941	5.00E−05	0.000208859
ENSG00000115263	GCG|glucagon	19.8941	5.00E−05	0.000208859
ENSG00000124731	TREM1|triggering receptor expressed on myeloid cells 1	19.8941	5.00E−05	0.000208859
ENSG00000131203	IDO1|indoleamine 2,3-dioxygenase 1	19.8941	5.00E−05	0.000208859
ENSG00000132185	FCRLA|Fc receptor-like A	19.8941	5.00E−05	0.000208859
ENSG00000133665	DYDC2|DPY30 domain containing 2	19.8941	5.00E−05	0.000208859
ENSG00000137491	SLCO2B1|solute carrier organic anion transporter family, member 2B1	19.8941	5.00E−05	0.000208859
ENSG00000142583	SLC2A5|solute carrier family 2 (facilitated glucose/fructose transporter), member 5	19.8941	5.00E−05	0.000208859
ENSG00000145908	ZNF300|zinc finger protein 300	19.8941	5.00E−05	0.000208859
ENSG00000152315	KCNK13|potassium channel, two pore domain subfamily K, member 13	19.8941	5.00E−05	0.000208859
ENSG00000156219	ART3|ADP-ribosyltransferase 3	19.8941	5.00E−05	0.000208859
ENSG00000157093	LYZL4|lysozyme-like 4	19.8941	5.00E−05	0.000208859
ENSG00000160307	S100B|S100 calcium binding protein B	19.8941	5.00E−05	0.000208859
ENSG00000162723	SLAMF9|SLAM family member 9	19.8941	5.00E−05	0.000208859
ENSG00000165606	DRGX|dorsal root ganglia homeobox	19.8941	5.00E−05	0.000208859
ENSG00000165810	BTNL9|butyrophilin-like 9	19.8941	5.00E−05	0.000208859
Down-regulated				
ENSG00000069482	GAL|galanin/GMAP prepropeptide	− 15.6345	5.00E−05	0.000208859
ENSG00000116824	CD2|CD2 molecule	− 15.6345	5.00E−05	0.000208859
ENSG00000174899	PQLC2L|PQ loop repeat containing 2-like	− 15.6345	5.00E−05	0.000208859
ENSG00000236816	ANKRD20A7P|	− 15.6345	5.00E−05	0.000208859
ENSG00000271620	IGHV3OR16-7|	− 15.6345	5.00E−05	0.000208859
ENSG00000274391	TPTE|transmembrane phosphatase with tensin homology	− 15.6345	5.00E−05	0.000208859
ENSG00000064692	SNCAIP|synuclein, alpha interacting protein	− 5.55951	5.00E−05	0.000208859
ENSG00000149970	CNKSR2|connector enhancer of kinase suppressor of Ras 2	− 5.32997	5.00E−05	0.000208859
ENSG00000238266	LINC00707|long intergenic non-protein coding RNA 707	− 5.09575	5.00E−05	0.000208859
ENSG00000118513	MYB|v-myb avian myeloblastosis viral oncogene homolog	− 4.9925	5.00E−05	0.000208859
ENSG00000128052	KDR|kinase insert domain receptor	− 4.91021	5.00E−05	0.000208859
ENSG00000132182	NUP210|nucleoporin 210 kDa	− 4.88683	5.00E−05	0.000208859
ENSG00000158089	GALNT14|polypeptide N-acetylgalactosaminyltransferase 14	− 4.71677	5.00E−05	0.000208859
ENSG00000152128	TMEM163|transmembrane protein 163	− 4.71169	5.00E−05	0.000208859
ENSG00000175352	NRIP3|nuclear receptor interacting protein 3	− 4.43541	5.00E−05	0.000208859
ENSG00000159708	LRRC36|leucine rich repeat containing 36	− 4.39752	5.00E−05	0.000208859
ENSG00000168280	KIF5C|kinesin family member 5C	− 4.37861	5.00E−05	0.000208859
ENSG00000166669	ATF7IP2|activating transcription factor 7 interacting protein 2	− 4.36058	5.00E−05	0.000208859
ENSG00000177359	|ovostatin 2///|ovostatin	− 4.26232	5.00E−05	0.000208859
ENSG00000249628	LINC00942|	− 4.06735	5.00E−05	0.000208859

### KEGG annotation of DEG and enriched biological processes analysis

**Fig. 2:**
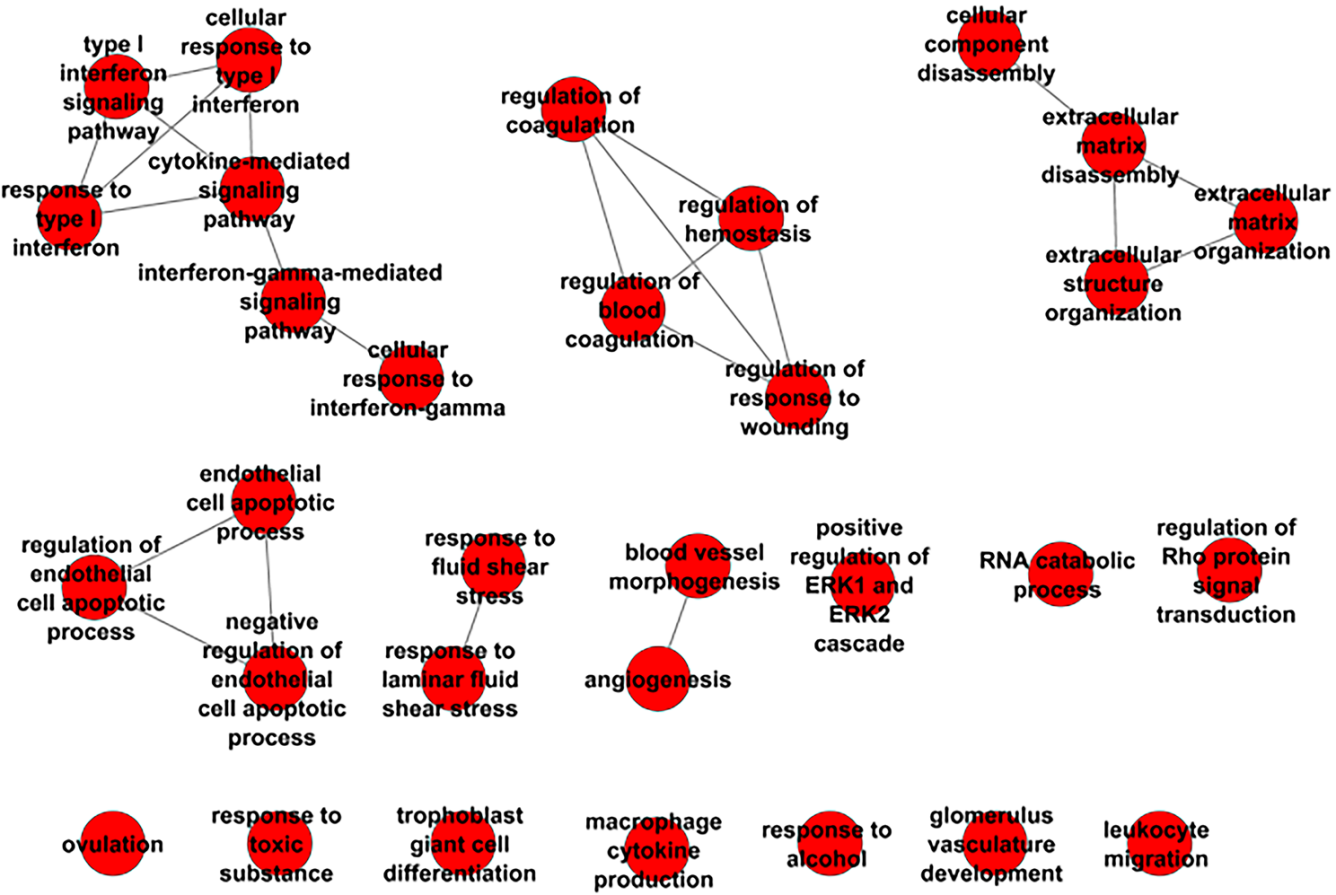
Enriched biological processes (BP) analysis of ER− palbociclib-resistant breast cancer cells

To identify the underlying molecular mechanisms driving resistance to palbociclib, we performed KEGG pathway analysis of all DEGs identified using CategoryCompare [23]. Table 2 lists the enriched KEGG pathways identified in 231/pR vs. 231/pS cells (false discovery rate (FDR) ≤ 0.05 and *p*-value ≤ 0.001). The KEGG terms associated with resistance to palbociclib included ‘ECM–receptor interaction and ‘Focal adhesion’. Next, we sought to identify biological processes that correlated with palbociclib-resistance by performing analysis of GO:BP (Fig. 2). We observed distinct groups of nodes including ‘type I interferon signaling’, ‘extracellular matrix assembly and organization’, ‘angiogenesis’, ‘regulation of endothelial cells’, ‘regulation of ERK signaling’ and ‘regulation of Rho signaling’ revealing multiple functional “themes” associated with resistance to palbociclib.

**Table 2:** Top enriched KEGG terms between 231/pS and 231/pR ranked by *p*-value (*p*_val_ ≤ 0.05; *q*_val_ ≤ 0.05; |log_2_FC|≥ 1)

KEGG ID	Description	*p*-value	FDR
5150	*Staphylococcus aureus* infection	3.19E−05	0
4512	ECM–receptor interaction	7.69E−05	0
5323	Rheumatoid arthritis	9.74E−05	0.006666667
4610	Complement and coagulation cascades	3.30E−04	0.005

### Canonical pathway analysis of DEG

**Fig. 3:**
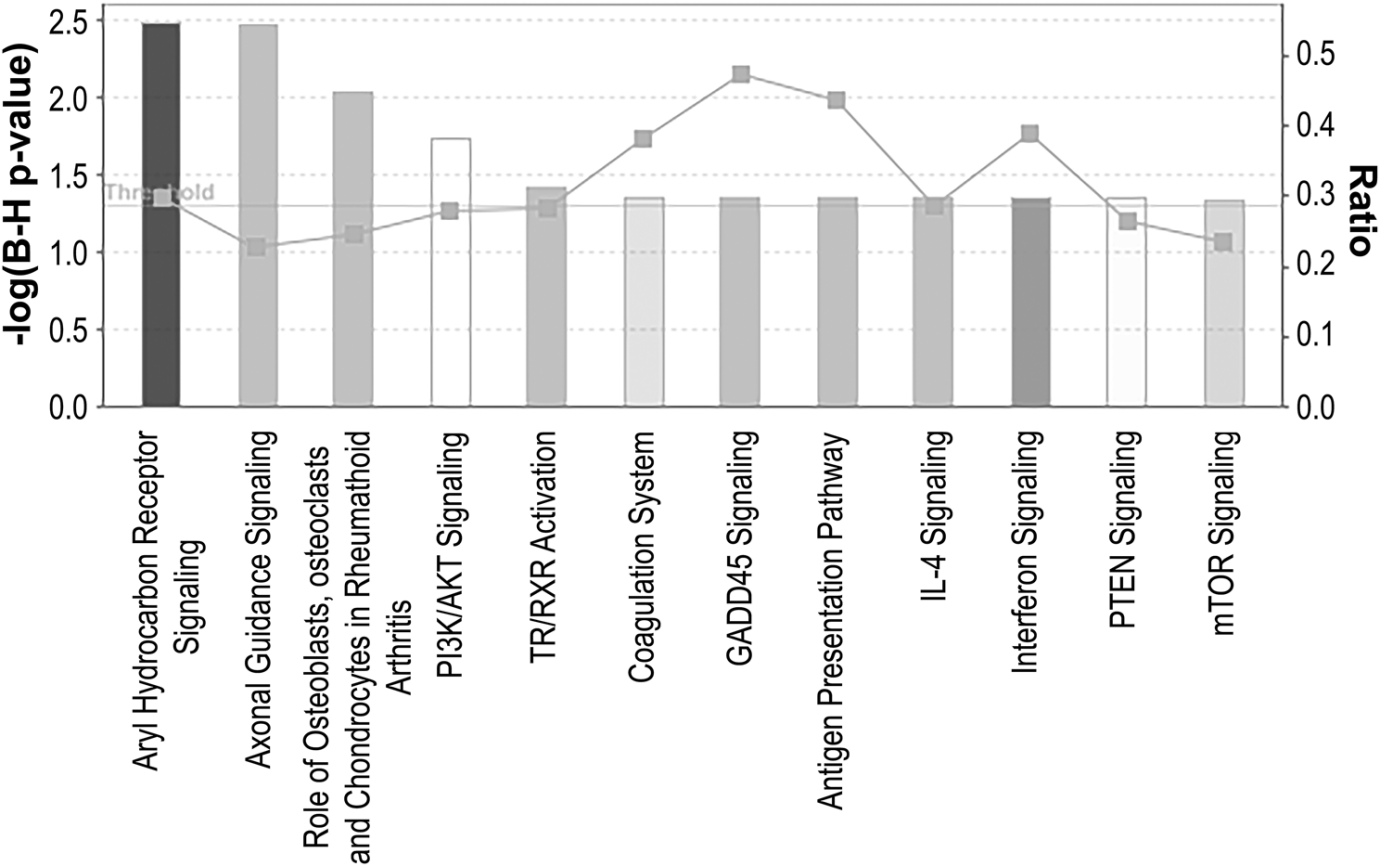
IPA analysis of ER− palbociclib-resistant breast cancer cells. A higher −log(B–H *p*-value) shown on the left *Y* axis represents more significant pathways. The ratio (right *Y* axis) refers to the number of genes from the dataset that map to the pathway divided by the total number of genes that map the canonical pathway from the IPA database. *p*_val_ ≤ 0.05; *q*_val_ ≤ 0.05; |log_2_FC|≥ 1

To uncover pathways significantly associated with resistance to palbociclib, all altered transcripts were mapped to known pathways using Ingenuity Pathway Analysis (IPA). The canonical pathway ‘Aryl hydrocarbon receptor’ emerged as the most significantly associated with resistance to palbociclib. Additionally, we observed significant enrichment of several canonical pathways including three pathways involved in immune responses (‘Antigen presentation pathway’, ‘IL-4 signaling’ and ‘Interferon signaling’), three pathways involved in signal transduction (‘PI3K/AKT signaling’, ‘PTEN signaling and ‘mTOR signaling’), among others (Fig. 3).

### Metabolic pathways analysis of DEGs

**Fig. 4:**
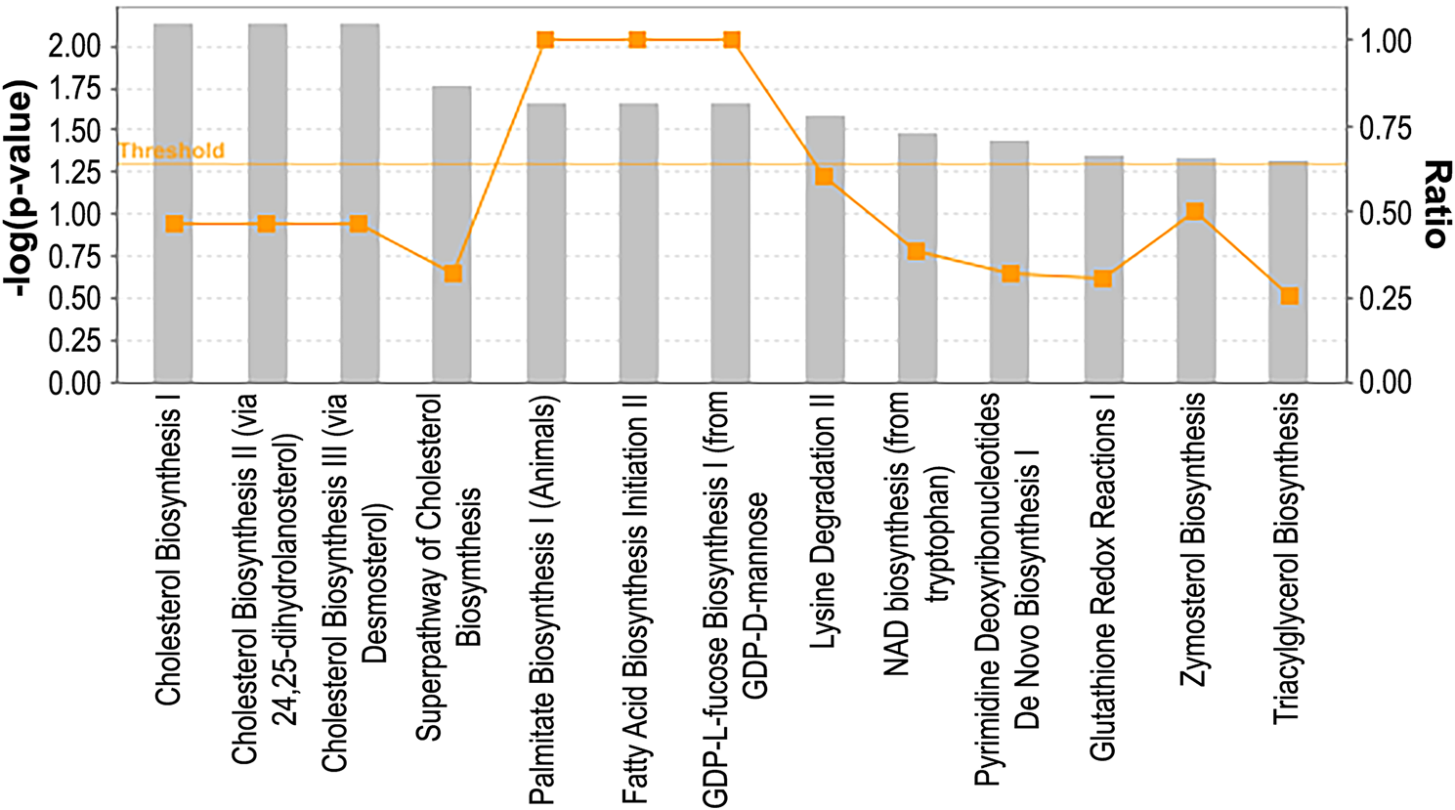
Metabolic pathway analysis of ER− palbociclib-resistant breast cancer cells. A higher −log(*p*-value) shown on the left *Y* axis represents more significant pathways. The ratio (right *Y* axis) refers to the number of genes from the dataset that map to the pathway divided by the total number of genes that map the canonical pathway from the IPA database. *p*_val_ ≤ 0.05; *q*_val_ ≤ 0.05; |log_2_FC|≥ 1

We next performed metabolic pathway analysis of all DEGs using IPA with the goals of determining the underlying metabolic differences between 231/pR and 231/pS cells (Fig. 4). We observed a significant enrichment of metabolic pathways involved in cholesterol biosynthesis (‘Cholesterol biosynthesis I’, ‘Cholesterol Biosynthesis II’ and ‘Superpathway of cholesterol biosynthesis’) and fatty acid biosynthesis (‘Fatty acid biosynthesis’, ‘Palmitate biosynthesis I’ and ‘Triacylglycerol biosynthesis’). Among other pathways, we found ‘NAD biosynthesis (from Tryptophan)’ and ‘Pyrimidine deoxyribonucleotides de novo biosynthesis I’ to be enriched in our dataset. These results indicate that deregulated metabolism, particularly cholesterol and fatty acid metabolism, may play an essential role in mediating resistance to palbociclib.

### Validation of expression of DEGs identified by RNA-seq

**Fig. 5:**
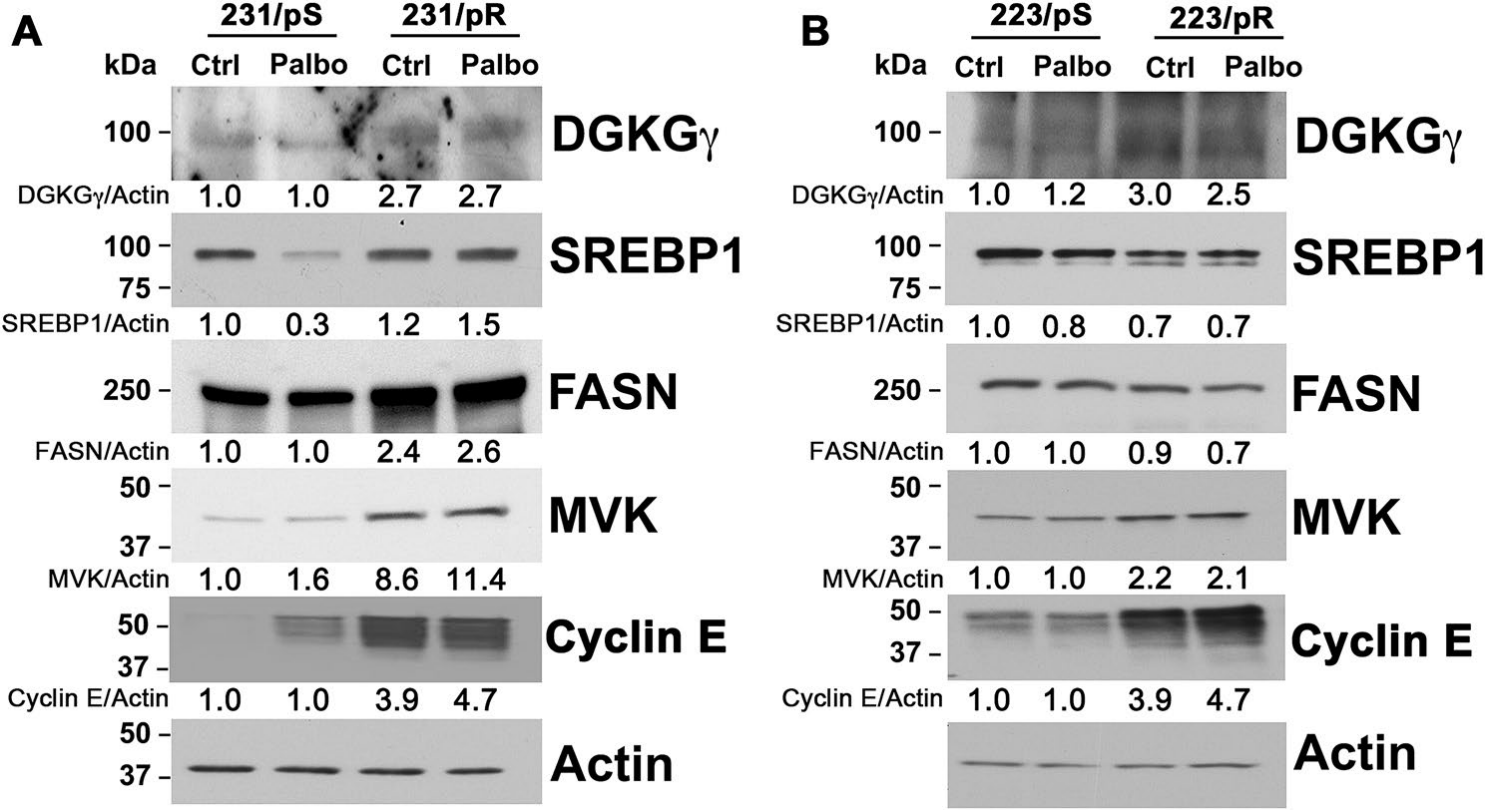
Protein expression correlates with DEGs identified by RNA-seq. Cells were treated with either vehicle control (Ctrl) or 500 nM palbociclib (Palbo), harvested after 24 h and immunoblotted with the indicated antibodies. **a** MDA-MB-231 cells. **b** MFM-223 cells. Quantitative densitometry analysis is shown relative to the actin protein normalized to palbociclib-sensitive control-treated cells

To validate our RNA-seq data, we selected a number of DEGs with potential biological relevance (as identified by our IPA analysis) to determine their expression at the protein level in 231/pS and 231/pR cells exposed to either vehicle control or palbociclib. Given that DGKGγ was identified as one of the top 20 up-regulated genes in our dataset (Table 1) we next examined DGKGγ protein expression in these cells. Diacylglycerol kinase gamma (DGKGγ) belongs to a family of DGKGs that convert diacylglycerol to phosphatidic acid thus, regulating the levels of two lipids with key roles in cell signaling and metabolism. We observed a marked increase in DGKGγ protein expression in 231/pR cells compared to 231/pS. Treatment with palbociclib had no effect on DGKGγ protein expression compared to the corresponding vehicle control (Fig. 5a). Our metabolic pathway analysis identified an enrichment in fatty acid and cholesterol biosynthesis in 231/pR vs. 231/pS cells. Lipid metabolism is tightly regulated by sterol regulatory element-binding factors (SREBPs), which activate the transcription of genes encoding for key enzymes involved in fatty acid and cholesterol biosynthesis [25, 26]. Protein expression analysis of SREBP1 showed a modest increase in SREBP1 in 231/pR control-treated cells relative to 231/pS (Fig. 5a). Notably, while palbociclib treatment in 231/pS cells led to a marked decrease in SREBP1 expression, 231/pR cells failed to decrease SREBP1 expression in response to palbociclib exposure. Next, we examined the expression of fatty acid synthase (FASN), a key enzyme regulating fatty acid synthesis and a direct target of SREBP1. We found that FASN protein levels were significantly increased in 231/pR cells compared to 231/pS cells in the presence and absence of palbociclib. Mevalonate kinase (MVK) is an essential early enzyme in the mevalonate pathway which is required for the generation of isoprenoids and cholesterol. We observed a marked increase in MVK in 231/pR cells compared to 231/pS cells in the presence and absence of palbociclib. Given the known heterogeneity of TNBC, we next sought to determine whether the observed changes in gene expression upon the development of resistance were cell-type specific. We used MFM-223 cells, which are sensitive to palbociclib, and generated palbociclib-resistant MFM-223 cells (223/pR) by continuous exposure to palbociclib for 4 months. Protein expression analysis of 223/pR cells showed an increase in DGKGγ and MVK levels compared to 223/pS cells. In contrast, a modest decrease was observed in the expression levels of SREBP1 and FASN in 223/pR cells compared to the parental 223/pS cells. These observations suggest that DGKGγ and MVK may be key mediators of resistance to palbociclib. In line with a previous study, the 223/pR cells acquired increased expression of cyclin E which is a known mechanism of resistance to CDK4/6 inhibition (Fig. 5b) [17]. This increase in cyclin E was also observed in 231/pR cells compared to 231/pS (Fig. 5a).

Taken together, our results validate the expression of various DEGs identified in our RNA-seq analysis. Furthermore, our findings suggest an up-regulation of *cholesterol biosynthesis in both TNBC models* that may mediate resistance to palbociclib.

## Discussion

A significant number of patients with TNBC experience disease progression within 2–5 years from initial diagnosis. To benefit these patients, we need to develop effective targeted therapies against this cancer subtype [27]. Given the success of CDK4/6 inhibitors in advanced ER+ breast cancer, trials are underway to expand the use of this treatment modality against TNBC. Resistance to CDK4/6 inhibition is a major limitation of this treatment regimen thus, a mechanistic understanding of CDK4/6 resistance is needed to design effective combinatorial therapies that block this adaptive response to CDK4/6 inhibition. In this study, we performed transcriptomic analysis to characterize a pair of palbociclib-sensitive and -resistant ER- MDA-MB-231 breast cancer cells. To the best of our knowledge, we are the first group to generate MDA-MB-231 cells with acquired resistance to palbociclib and to identify clinically relevant pathways associated with resistance in these cells.

Examination of canonical pathways associated with resistance to palbociclib in ER− breast cancer cells revealed a deregulation of PI3K/AKT/mTOR signaling (Fig. 3). These results are in line with previous studies showing that PI3K/AKT/mTOR activation mediates resistance to CDK4/6 inhibition in ER+ breast cancer [28]. Importantly, the PI3K/AKT/mTOR axis play a critical role in regulating cellular metabolism including lipid metabolism [29, 30] and may be responsible for the deregulation of fatty acid and cholesterol observed in palbociclib-resistant TNBC cells. Similarly, we observed an increase in cyclin E in both TNBC cell models of acquired resistance to palbociclib. The role of cyclin E in mediating resistance to CDK4/6 inhibition has been extensively documented in ER+ breast cancer cells. Taken together, our results suggest that reactivation of the PI3K/AKT/mTOR pathway and increased cyclin E are universal mechanisms of resistance to CDK4/6 inhibition independent of ER status.

Metabolic rewiring is consistently observed in cancer cells and is an emergent mechanism of resistance to CDK4/6 inhibition. For instance, a previous study showed that palbociclib-resistant ER+/HER2− breast cancer cells exhibit increased dependency in glucose metabolism [31]. In this context, elucidating the unique metabolic dependencies of sensitive versus therapy-resistant cells remains an important challenge that may reveal novel pharmacological interventions. Our transcriptomic analysis uncovered that fatty acid and cholesterol metabolism are the most common metabolic alterations in 231/pR compared to 231/pS cells, suggesting that lipid reprogramming is associated with resistance to palbociclib. Here, we show that 231/pR cells increase the expression of important drivers of fatty acid and cholesterol metabolisms such as SREBP1, FASN and MVK (Fig. 5a). Notably, MVK expression was also found to be increased in 223/pR cells compared to 223/pS cells, suggesting that MVK may be a key mediator of resistance to palbociclib across multiple TNBC cells. Conversely, we observed a modest decrease in SREBP1 and FASN in 223/pR cells compared to 223/pS. It should be noted that other drivers of fatty acid biosynthesis include key enzymes such as acetyl CoA carboxylase (ACC) and ATP citrate lyase (ACLY) [32, 33], thus additional studies will be required to determine if increased fatty acid metabolism occurs in 223/pR cells via upregulation of these enzymes. Lipid metabolic rewiring is an emerging mechanism of resistance to many targeted therapies in breast cancer [34]. Several groups have demonstrated that lipid reprogramming promotes the growth and survival of cancer cells and often correlates with cancer cell stemness and resistance to therapy [35–40]. Moreover, elevated total cholesterol levels have been shown to correlate with early recurrence in breast cancer [41] while the use of cholesterol-lowering medications associates with improved overall survival in a subset of TNBC patients and decreases the survival of TNBC cell lines [42, 43]. Our studies provide further evidence of the potential role of lipid metabolism deregulation in mediating resistance to CDK4/6 inhibition and provide rationale for the future testing of drugs targeting lipid metabolism to re-sensitize TNBC tumors resistant to CDK4/6 inhibitors.

Our findings indicate that cholesterol metabolism was significantly altered in 231/pR and 223/pR cells compared their respective sensitive counterpart. Future metabolomic profiling will be needed to confirm our initial findings and provide further evidence as to whether cholesterol-lowering drugs such as statins may provide anticancer activity against palbociclib-resistant TNBC. Importantly, while resistance to palbociclib has been associated with changes in glucose and nucleotide metabolism in ER+ breast cancer cells [31, 44], the metabolic changes herein described have not been reported in ER+ breast cancer cells thus far. Given the heterogeneity of TNBC, a limitation of our studies is the use of only two isogenic TNBC cell lines to characterize the mechanisms of resistance to palbociclib. Future studies will be needed to determine whether our findings extend across multiple TNBC subtypes and to functionally validate whether these metabolic alterations may have potential clinical utility. Another limitation of the current analysis is the use of cells that are resistant to a single CDK4/6 inhibitor, palbociclib. Preclinical evidence in some ER+ models suggests that resistance to palbociclib confers cross-resistance to the other CDK4/6 inhibitors and thus, it is likely that a number of mechanisms of resistance described here will be shared with other CDK4/6 inhibitors [45]. Nevertheless, the data presented here will help guide future efforts to study these potentially targetable alterations in response to CDK4/6 inhibition in TNBC.

Collectively, our studies identified clinically relevant canonical and metabolic pathways associated with resistance to palbociclib in ER-MDA-MB-231 cells. Future studies will focus on characterizing the dependency of TNBC resistant to CDK4/6 inhibitors on fatty acid and cholesterol reprograming and determine the utility of targeting key enzymes such as MVK for the prevention and/or treatment of drug-resistant TNBC patients.

## Supplementary Information

Below is the link to the electronic supplementary material.

Supplementary file1 (XLSX 6472 KB)

## Abbreviations

TNBC: Triple-negative breast cancer
KEGG: Kyoto encyclopedia of genes and genomes
ER: Estrogen receptor
PR: Progesterone receptor
CDK4/6: Cyclin-dependent kinases 4 and 6
DEG: Differentially expressed genes
IPA: Ingenuity pathway analysis

## References

[1] Foulkes WD, Smith IE, Reis-Filho JS (2010). Triple-negative breast cancer. N Engl J Med.

[2] Rastelli F, Biancanelli S, Falzetta A, Martignetti A, Casi C, Bascioni R, Giustini L, Crispino S (2010). Triple-negative breast cancer: current state of the art. Tumori.

[3] Metzger-Filho O, Tutt A, de Azambuja E, Saini KS, Viale G, Loi S, Bradbury I, Bliss JM, Azim HA, Ellis P (2012). Dissecting the heterogeneity of triple-negative breast cancer. J Clin Oncol.

[4] Gupta GK, Collier AL, Lee D, Hoefer RA, Zheleva V, Siewertsz van Reesema LL, Tang-Tan AM, Guye ML, Chang DZ, Winston JS (2020). Perspectives on triple-negative breast cancer: current treatment strategies, unmet needs, and potential targets for future therapies. Cancers (Basel).

[5] Surveillance E, End Results (SEER) Program (www.seer.cancer.gov) SEER*Stat Database: Incidence-SEER Research Data, 9 Registries, Nov 2019 Sub (1975–2017)-Linked To County Attributes-Time Dependent (1990–2017) Income/Rurality, 1969–2017 Counties, National Cancer Institute, DCCPS, Surveillance Research Program, released April 2020, based on the November 2019 submission

[6] Miller KD, Nogueira L, Mariotto AB, Rowland JH, Yabroff KR, Alfano CM, Jemal A, Kramer JL, Siegel RL (2019). Cancer treatment and survivorship statistics, 2019. CA Cancer J Clin.

[7] DeMichele A, Clark AS, Tan KS, Heitjan DF, Gramlich K, Gallagher M, Lal P, Feldman M, Zhang P, Colameco C (2015). CDK 4/6 inhibitor palbociclib (PD0332991) in Rb+ advanced breast cancer: phase II activity, safety, and predictive biomarker assessment. Clin Cancer Res.

[8] Schwartz GK, LoRusso PM, Dickson MA, Randolph SS, Shaik MN, Wilner KD, Courtney R, O'Dwyer PJ (2011). Phase I study of PD 0332991, a cyclin-dependent kinase inhibitor, administered in 3-week cycles (Schedule 2/1). Br J Cancer.

[9] VanArsdale T, Boshoff C, Arndt KT, Abraham RT (2015). Molecular pathways: targeting the cyclin D-CDK4/6 axis for cancer treatment. Clin Cancer Res.

[10] Dhillon S (2015). Palbociclib: first global approval. Drugs.

[11] Hortobagyi GN, Stemmer SM, Burris HA, Yap YS, Sonke GS, Paluch-Shimon S, Campone M, Blackwell KL, Andre F, Winer EP (2016). Ribociclib as first-line therapy for HR-positive, advanced breast cancer. N Engl J Med.

[12] Sobhani N, D’Angelo A, Pittacolo M, Roviello G, Miccoli A, Corona SP, Bernocchi O, Generali D, Otto T (2019). Updates on the CDK4/6 inhibitory strategy and combinations in breast cancer. Cells.

[13] Finn RS, Dering J, Conklin D, Kalous O, Cohen DJ, Desai AJ, Ginther C, Atefi M, Chen I, Fowst C (2009). PD 0332991, a selective cyclin D kinase 4/6 inhibitor, preferentially inhibits proliferation of luminal estrogen receptor-positive human breast cancer cell lines in vitro. Breast Cancer Res.

[14] Herschkowitz JI, He X, Fan C, Perou CM (2008). The functional loss of the retinoblastoma tumour suppressor is a common event in basal-like and luminal B breast carcinomas. Breast Cancer Res.

[15] Yamamoto T, Kanaya N, Somlo G, Chen S (2019). Synergistic anti-cancer activity of CDK4/6 inhibitor palbociclib and dual mTOR kinase inhibitor MLN0128 in pRb-expressing ER-negative breast cancer. Breast Cancer Res Treat.

[16] Yuan Y, Wen W, Yost SE, Xing Q, Yan J, Han ES, Mortimer J, Yim JH (2019). Combination therapy with BYL719 and LEE011 is synergistic and causes a greater suppression of p-S6 in triple negative breast cancer. Sci Rep.

[17] Asghar US, Barr AR, Cutts R, Beaney M, Babina I, Sampath D, Giltnane J, Lacap JA, Crocker L, Young A (2017). Single-cell dynamics determines response to CDK4/6 inhibition in triple-negative breast cancer. Clin Cancer Res.

[18] Teo ZL, Versaci S, Dushyanthen S, Caramia F, Savas P, Mintoff CP, Zethoven M, Virassamy B, Luen SJ, McArthur GA (2017). Combined CDK4/6 and PI3Kalpha inhibition is synergistic and immunogenic in triple-negative breast cancer. Cancer Res.

[19] Liu T, Yu J, Deng M, Yin Y, Zhang H, Luo K, Qin B, Li Y, Wu C, Ren T (2017). CDK4/6-dependent activation of DUB3 regulates cancer metastasis through SNAIL1. Nat Commun.

[20] Cretella D, Fumarola C, Bonelli M, Alfieri R, La Monica S, Digiacomo G, Cavazzoni A, Galetti M, Generali D, Petronini PG (2019). Pre-treatment with the CDK4/6 inhibitor palbociclib improves the efficacy of paclitaxel in TNBC cells. Sci Rep.

[21] Zhang J, Wang Q, Wang Q, Cao J, Sun J, Zhu Z (2020). Mechanisms of resistance to estrogen receptor modulators in ER+/HER2- advanced breast cancer. Cell Mol Life Sci.

[22] Illumina I (2014) BaseSpace User Guide, vol 15044182 Rev. E, pp 1–98

[23] Flight RM, Harrison BJ, Mohammad F, Bunge MB, Moon LD, Petruska JC, Rouchka EC (2014). categoryCompare, an analytical tool based on feature annotations. Front Genet.

[24] Lypova N, Lanceta L, Gibson A, Vega S, Garza-Morales R, McMasters KM, Chesney J, Gomez-Gutierrez JG, Imbert-Fernandez Y (2019). Targeting palbociclib-resistant estrogen receptor-positive breast cancer cells via oncolytic virotherapy. Cancers (Basel).

[25] Horton JD, Goldstein JL, Brown MS (2002). SREBPs: activators of the complete program of cholesterol and fatty acid synthesis in the liver. J Clin Investig.

[26] Ye J, DeBose-Boyd RA (2011). Regulation of cholesterol and fatty acid synthesis. Cold Spring Harb Perspect Biol.

[27] Tan DS, Marchio C, Jones RL, Savage K, Smith IE, Dowsett M, Reis-Filho JS (2008). Triple negative breast cancer: molecular profiling and prognostic impact in adjuvant anthracycline-treated patients. Breast Cancer Res Treat.

[28] Herrera-Abreu MT, Palafox M, Asghar U, Rivas MA, Cutts RJ, Garcia-Murillas I, Pearson A, Guzman M, Rodriguez O, Grueso J (2016). Early adaptation and acquired resistance to CDK4/6 inhibition in estrogen receptor-positive breast cancer. Cancer Res.

[29] Mao Z, Zhang W (2018). Role of mTOR in glucose and lipid metabolism. Int J Mol Sci.

[30] Furuta E, Pai SK, Zhan R, Bandyopadhyay S, Watabe M, Mo YY, Hirota S, Hosobe S, Tsukada T, Miura K (2008). Fatty acid synthase gene is up-regulated by hypoxia via activation of Akt and sterol regulatory element binding protein-1. Cancer Res.

[31] Lorito N, Bacci M, Smiriglia A, Mannelli M, Parri M, Comito G, Ippolito L, Giannoni E, Bonechi M, Benelli M (2020). Glucose metabolic reprogramming of ER breast cancer in acquired resistance to the CDK4/6 inhibitor palbociclib+. Cells.

[32] Yoon S, Lee MY, Park SW, Moon JS, Koh YK, Ahn YH, Park BW, Kim KS (2007). Up-regulation of acetyl-CoA carboxylase alpha and fatty acid synthase by human epidermal growth factor receptor 2 at the translational level in breast cancer cells. J Biol Chem.

[33] Wang D, Yin L, Wei J, Yang Z, Jiang G (2017). ATP citrate lyase is increased in human breast cancer, depletion of which promotes apoptosis. Tumour Biol.

[34] Feng WW, Kurokawa M (2020). Lipid metabolic reprogramming as an emerging mechanism of resistance to kinase inhibitors in breast cancer. Cancer Drug Resist.

[35] Pavlova NN, Thompson CB (2016). The emerging hallmarks of cancer metabolism. Cell Metab.

[36] Beloribi-Djefaflia S, Vasseur S, Guillaumond F (2016). Lipid metabolic reprogramming in cancer cells. Oncogenesis.

[37] Liu Q, Luo Q, Halim A, Song G (2017). Targeting lipid metabolism of cancer cells: a promising therapeutic strategy for cancer. Cancer Lett.

[38] Wang T, Fahrmann JF, Lee H, Li YJ, Tripathi SC, Yue C, Zhang C, Lifshitz V, Song J, Yuan Y (2018). JAK/STAT3-regulated fatty acid beta-oxidation is critical for breast cancer stem cell self-renewal and chemoresistance. Cell Metab.

[39] Chen CL, Uthaya Kumar DB, Punj V, Xu J, Sher L, Tahara SM, Hess S, Machida K (2016). NANOG metabolically reprograms tumor-initiating stem-like cells through tumorigenic changes in oxidative phosphorylation and fatty acid metabolism. Cell Metab.

[40] Feng WW, Wilkins O, Bang S, Ung M, Li J, An J, Del Genio C, Canfield K, DiRenzo J, Wells W (2019). CD36-mediated metabolic rewiring of breast cancer cells promotes resistance to HER2-targeted therapies. Cell Rep.

[41] Bahl M, Ennis M, Tannock IF, Hux JE, Pritchard KI, Koo J, Goodwin PJ (2005). Serum lipids and outcome of early-stage breast cancer: results of a prospective cohort study. Breast Cancer Res Treat.

[42] Shaitelman SF, Stauder MC, Allen P, Reddy S, Lakoski S, Atkinson B, Reddy J, Amaya D, Guerra W, Ueno N (2017). Impact of statin use on outcomes in triple negative breast cancer. J Cancer.

[43] Park YH, Jung HH, Ahn JS, Im YH (2013). Statin induces inhibition of triple negative breast cancer (TNBC) cells via PI3K pathway. Biochem Biophys Res Commun.

[44] Lanceta L, O’Neill C, Lypova N, Li X, Rouchka E, Waigel S, Gomez-Gutierrez JG, Chesney J, Imbert-Fernandez Y (2020). Transcriptomic profiling identifies differentially expressed genes in palbociclib-resistant ER+ MCF7 breast cancer cells. Genes (Basel).

[45] Ogata R, Kishino E, Saitoh W, Koike Y, Kurebayashi J (2021). Resistance to cyclin-dependent kinase (CDK) 4/6 inhibitors confers cross-resistance to other CDK inhibitors but not to chemotherapeutic agents in breast cancer cells. Breast Cancer.

